# Post-Translational Modifications of Proteins Orchestrate All Hallmarks of Cancer

**DOI:** 10.3390/life15010126

**Published:** 2025-01-18

**Authors:** Pathea Shawnae Bruno, Aneeta Arshad, Maria-Raluca Gogu, Natalie Waterman, Rylie Flack, Kimberly Dunn, Costel C. Darie, Anca-Narcisa Neagu

**Affiliations:** 1 Biochemistry & Proteomics Laboratories, Department of Chemistry and Biochemistry, Clarkson University, Potsdam, NY 13699-5810, USA; brunop@clarkson.edu (P.S.B.); anarsha@clarkson.edu (A.A.); watermnd@clarkson.edu (N.W.); flackrn@clarkson.edu (R.F.); dunnks@clarkson.edu (K.D.); 2 Advanced Research and Development Center for Experimental Medicine (CEMEX), “Grigore T. Popa” University of Medicine and Pharmacy, University Street No. 16, 700115 Iasi, Romania; raluca.gogu@umfiasi.ro; 3 Laboratory of Animal Histology, Faculty of Biology, “Alexandru Ioan Cuza” University of Iași, Carol I bvd. 20A, 700505 Iasi, Romania

**Keywords:** cancer, hallmarks, enabling characteristics, post-translational modifications of proteins (PTMs), protein subcellular localization, protein activity

## Abstract

Post-translational modifications (PTMs) of proteins dynamically build the buffering and adapting interface between oncogenic mutations and environmental stressors, on the one hand, and cancer cell structure, functioning, and behavior. Aberrant PTMs can be considered as enabling characteristics of cancer as long as they orchestrate all malignant modifications and variability in the proteome of cancer cells, cancer-associated cells, and tumor microenvironment (TME). On the other hand, PTMs of proteins can enhance anticancer mechanisms in the tumoral ecosystem or sustain the beneficial effects of oncologic therapies through degradation or inactivation of carcinogenic proteins or/and activation of tumor-suppressor proteins. In this review, we summarized and analyzed a wide spectrum of PTMs of proteins involved in all regulatory mechanisms that drive tumorigenesis, genetic instability, epigenetic reprogramming, all events of the metastatic cascade, cytoskeleton and extracellular matrix (ECM) remodeling, angiogenesis, immune response, tumor-associated microbiome, and metabolism rewiring as the most important hallmarks of cancer. All cancer hallmarks develop due to PTMs of proteins, which modulate gene transcription, intracellular and extracellular signaling, protein size, activity, stability and localization, trafficking, secretion, intracellular protein degradation or half-life, and protein–protein interactions (PPIs). PTMs associated with cancer can be exploited to better understand the underlying molecular mechanisms of this heterogeneous and chameleonic disease, find new biomarkers of cancer progression and prognosis, personalize oncotherapies, and discover new targets for drug development.

## 1. Introduction

Even if cancer is considered a genetic disease, it may have its origin in epigenetic processes and could be a consequence of stress-buffering mechanisms that allow cells to survive [1]. Post-translational modifications (PTMs) of proteins occur through the addition of various chemical groups, such as phosphoryl, acetyl, or methyl, or small proteins/carbohydrates/lipids, to one or more amino acid residues or result from the conversion of a residue to another, and are essential to maintain homeostasis of cells and tissues, but are also common in human cancer [2]. PTMs modulate protein stability, localization, activity, protein–protein interactions (PPIs), and half-life to cope with changing environmental conditions and cellular stress [3,4,5]. Thus, PTMs increase proteomic diversity, so the characterization of various functions of PTMs of proteins, as well as the understanding of modification dynamic and cooperation, is crucial in cancer as well as in any other disease [3,6]. Mitosis, DNA damage response (DDR), gene expression, cell cycle, cell signaling pathways, energy, apoptosis, and metabolic pathways are regulated by PTMs of histones and non-histone proteins [7]. Induced by stressful conditions, autophagy, a process that allows for the survival of cancer cells, causes PTMs of proteins, which are recognized by the immune system through a mechanism that can be used to enhance immune defense [8]. PTMs change the physical and chemical characteristics of proteins through conformational alterations, modify their stability and activity, subcellular localization, and protein–protein interactions (PPIs) [2]. Hundreds of PTMs of thousands of proteins have been reported [9,10], including chemical modifications (phosphorylation, acetylation, methylation, and oxidation), polypeptide modifications (including ubiquitination, SUMOylation, and NEDDylation), modifications by complex molecules, such as glycans, lipids and glycosaminoglycans, and modifications of the amino acids or the polypeptide backbone [11]. PTMs can be transient or stable in the cell [5].

The most studied functions of PTMs in cancer cells and TME are summarized in Table 1. Phosphorylation, one of the most important PTM of proteins, occurs by the attachment of a phosphate group to specific amino acid residues of a protein [12,13]. Ubiquitination (also named ubiquitylation or ubiquitinylation), the second most common PTM of proteins, covalently modifies substrate proteins with ubiquitin [14], mediating protein degradation and turnover [15]. SUMOylation involves the covalent attachment of a small ubiquitin-like modifier (SUMO) molecule to a substrate protein, regulating numerous cellular processes [16]. Protein acylation, especially responsive to metabolic changes in cancer, is also involved in protein stability, subcellular localization, enzyme activity, transcription, PPIs, and protein-DNA interactions, including acetylation [17,18] and novel forms of acylation [19], such as malonylation [20], crotonylation [21], succinylation [22], glutarylation, β-hydroxybutyrylation [23,24], dihidroxyisobutyrylation, benzoylation, lactylation, myristoylation, prenylation, and palmitoylation [25]. Acetylation, a PTM that participates in gene expression, cell cycle progress, DNA repair, and cancer cell proliferation, occurs by the transfer of acetyl groups from acetyl-coenzyme A to a specific site on a polypeptide chain [18,26]. Recently, Qin et al. (2024) showed that lysine β-hydroxybutyrylation is a PTM that can be induced by the ketogenic diet, reshaping cancer metabolism [23]. Glycosylation is a glucosyltransferase-dependent enzymatic reaction that involves the addition of individual carbohydrates or whole oligosaccharides (glycans) to corresponding protein or lipid [27]. NEDDylation consists of the attachment of a ubiquitin-like protein, the neural precursor cell expressed developmentally downregulated protein 8 (NEDD8), to a lysine residue of a protein substrate represented by cullins and non-cullins proteins [28]. S-nitrosylation, a PTM that consists of the addition of a nitrosyl group to the reactive thiol group of cysteine to form S-nitrosothiol, is involved in protein stability and turnover, steroid synthesis, transcription regulation, DDR, cellular growth, apoptosis, and redox regulation [29]. Protein citrullination/citrullinome is a rich source of neo-antigens in BC with applications for diagnosis and therapy [30].

## 2. PTMs’ Role in Cancer Hallmarks and Enabling Characteristics

Hanahan (2022) proposed an extended list of cancer hallmarks and enabling characteristics, including sustaining proliferative signaling, evading growth suppressors, nonmutational epigenetic reprogramming, avoiding immune destruction, enabling replicative immortality, tumor-promoting inflammation, polymorphic microbes, activating invasion and metastasis, inducing or accessing vasculature, senescent cells, genome instability and mutation, resisting cell death, deregulating cellular metabolism, and unlocking phenotypic plasticity [58]. In this review, we summarized and analyzed the most studied PTMs of proteins that orchestrate all cancer hallmarks.

### 2.1. PTMs and Cancer Hallmarks

#### 2.1.1. Sustaining Proliferative Signaling

**Figure 1:**
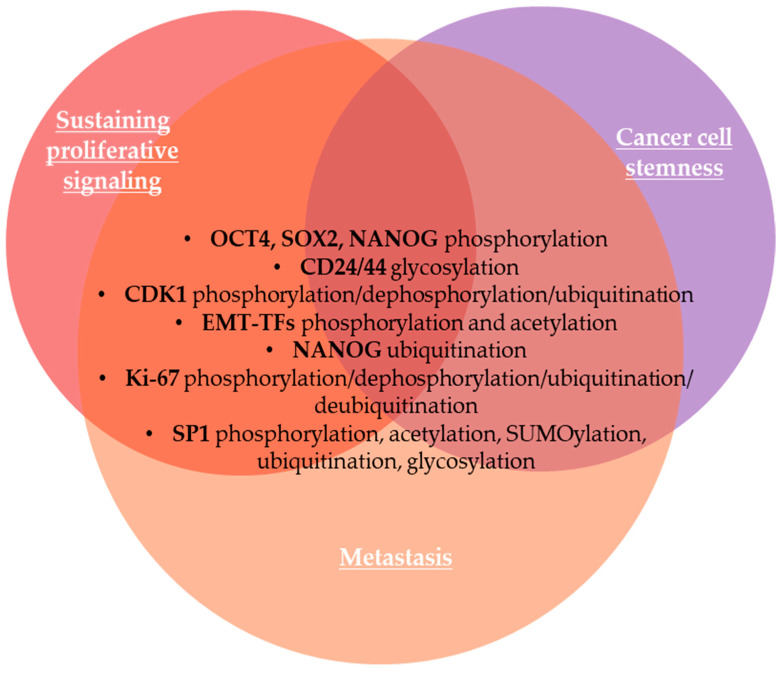
PTMs of protein biomarkers involved in sustaining proliferative signaling in association with cancer cell stemness and metastasis. CDK1—cyclin-dependent kinase 1; EMT-TFs—epithelial-to-mesenchymal transition-related transcription factors; Ki-67—marker of proliferation Kiel 67; OCT4—octamer-binding transcription factor 4; SOX2—SRY-box transcription factor 2; SP1—specific protein 1 transcription factor.

Proliferation, a crucial process in cancer development and progression, is widely associated with deregulation of cell cycle-associated proteins [59]. Hypoxia is also a hallmark of cancer [60] and a driving force for cancer cell proliferation and progression of tumors [61]. As a common hallmark of cancer, tumor cell populations maintain cell proliferation potential even in oxygen-deprivation conditions, mainly for the maintenance of cancer stem cells (CSCs) [62]. For various types of tumors, different patterns of PTMs of proteins, including phosphorylation, glycosylation, methylation, acetylation, ubiquitination, SUMOylation, lactylation, and palmitoylation, can modulate the cancer stemness (Figure 1), known as the ability of tumor cells to self-renew and differentiate, initiating tumor relapse and therapy resistance [63]. It is known that phosphorylation cascades play a central role in pluripotent cells, occurring in pluripotency factors such as octamer-binding transcription factor 4 (OCT4), SRY-box transcription factor 2 (SOX2), and NANOG [64]. Cluster of differentiation 24 (CD24) is a highly glycosylated cell surface adhesion protein and signaling molecule known to regulate CSC-like characteristics, emerging as a crucial CSC biomarker, overexpressed in various cancers such as lung, prostate, colon, pancreatic, and bladder tumors, among others, or contrarily, downregulated in breast cancer, leading to BCSCs proliferation and metastasis [65,66,67]. CD24 and CD44 surface markers regulate phosphorylation and acetylation of signal transducer and activator of transcription-3 (STAT3), maintaining stemness and EMT of cancer cells [68]. In addition, it was demonstrated that changes in CD44 glycosylation regulate its binding to hyaluronic acid, fibronectin, collagen, and podoplanin, and modulates c-Src/STAT3/TWIST1, PI3K/AKT/mTOR, ERK/NF-kB/NANOG and other signaling pathways, modulating TME traits and tumor cells behavior [69]. NANOG, a homeobox TF, is also a biomarker of tumor pluripotency in various cancers, such as ovarian, breast, and prostate cancers, with NANOG stability being assured through phosphorylation by ERK and cyclin-dependent kinase 1 (CDK1) [64,70]. The level, stability, and functions of NANOG are regulated by the ubiquitination [71].

It is known that cell cycle progression involves global changes in protein phosphorylation catalyzed by cyclin-dependent kinases (CDKs) [72], a class of serine/threonine kinases that regulate the cell cycle and modulate the development of diverse malignancies [73]. CDK1 is an oncogenic kinase necessary for mitotic entry, thus playing an important role in cell cycle regulation [74,75]. Phosphorylation is the most important PTM to control the activity of CDK1 in addition to its binding to cyclin B1, forming together the cyclin B1-CDK1 complex, also called the pre-mitosis promoting factor (pre-MPF) [76]. This pre-MPF is maintained in a cytosolic inactive state by phosphorylation of CDK1 at inhibitory Tyr14/15 residues [76]. The MPF becomes activated at the end of the G2 phase of the cell cycle by CDK1 dephosphorylation of Tyr14/15 and subsequent phosphorylation of the activator Thr161 residue [76]. Recently, Bu et al. (2024) found a novel peptide derived from the C-terminus of forkhead box M1 (FOXM1), named M1-20, that enhances the ubiquitination and degradation of CDK1 through proteasome pathway, with potential inhibitory effects for tumor cells [74]. FOXM1, an oncogenic TF, acts as an important regulator of G2/M-phase transition, mitosis, and DNA damage response [77]. FOXM1 is highly upregulated in BC, promoting tumorigenesis, cancer growth, and progression, and phosphorylation regulates FOXM1 subcellular distribution, PPIs, and partners of gene regulation [78]. Lin et al. (2024) have shown that CDK1 is highly overexpressed in endometrial carcinoma, enhancing cell survival, proliferation, invasion, and migration, inhibiting cell apoptosis, and inducing DNA damage dependent on the CDK1-mediated phosphorylation of kinesin family member C1 (KIFC1) and subsequent activation of PI3K/AKT pathway, known to be involved in cell division and proliferation related to tumor development [79].

Encoded by the *MKI67* gene, the expression of Ki-67, one of the most reliable immunohistochemical nuclear biomarkers used by histopathologists to identify proliferating cells throughout the cell cycle, is also a prognostic biomarker in the clinic, as in the case of invasive BCs among other malignancies [75,80,81]. Ki-67 is inactive in resting cells [82]. PTMs of Ki-67 are involved in the bidirectional redistribution of the protein from the interior of the nucleus to the periphery of the condensed chromosomes [82]. Ki-67 has many consensus target sequences for CDK1 phosphorylation and remains phosphorylated throughout mitosis, while dephosphorylation by protein phosphatase 1 (PP1) assures the reversal of CDK1 phosphorylation during mitotic exit [75]. In the nucleus of non-small cell lung cancer cells, Ki-67 and ubiquitin-specific processing protease (USP7) have been found to be highly expressed [81]. Thus, the overexpression of USP7 can promote cell proliferation by deubiquitinating Ki-67 protein [81]. The specificity protein 1 (SP1) is a TF that binds to the *MKI67* promoter region to regulate the expression of genes that promote cell cycle progression, including E2F that stimulates Ki-67 transcription [75]. SP1 stability and activity are modulated by diverse PTMs, such as phosphorylation, acetylation, SUMOylation, ubiquitination, and glycosylation [83]. SP1 is degraded in normal cells, but it is overexpressed in cancer cells, contributing to the proliferation and survival of cancer cells [84]. SP1 is phosphorylated by CDK1 at Thr 739, and p-SP1 reduces its DNA-binding ability and facilitates the chromatin condensation process during mitosis [84]. SP1 is dephosphorylated by protein phosphatase 2A (PP2A) at the end of mitosis and the beginning of interphase and returned to the chromatin [84]. Thus, cancer cell proliferation is modulated by SP1 translocation during the cell cycle progression, orchestrated by CDK1 and PP2A [84].

#### 2.1.2. Evading Growth Suppressors

The evasion of anti-growth signaling is an important hallmark of cancer cells [85]. Ruhul Amin et al. (2015) summarized the most important pathways involved in growth signaling, such as p53, phosphatase and tensin homolog (PTEN), retinoblastoma protein (RB/p16 pathway), Hippo, growth differentiation factor 15 (GDF15), Notch, insulin-like growth factor (IGF), and Krűppel-like factor 5 (KLF5) [85].

**Figure 2:**
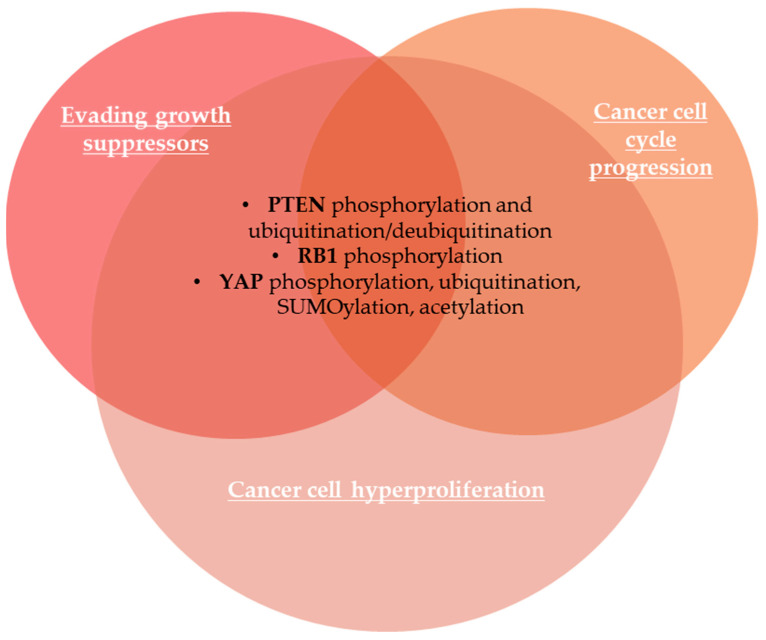
PTMs of biomarkers involved in evading growth suppressors in association with cancer cell cycle progression and cancer cell hyperproliferation. PTEN—phosphatase and tensin homolog deleted on chromosome 10; RB1—retinoblastoma protein 1; YAP1—YES-associated protein 1.

The tumor-suppressor PTEN, a phosphatidylinositol-3,4,5-triphosphate phospholipid phosphatase that catalyzes the dephosphorylation of phosphatidylinositol 3,4,5-triphosphate (PIP_3_) to phosphatidylinositol 4,5-diphosphate (PIP_2_) into the plasma membrane_,_ is important for cell growth signaling, being usually mutated or silenced in cancer cells [86]. PTEN inactivation leads to the activation of phosphoinositide 3-kinase (PI3K) and subsequent downstream AKT and mammalian target of rapamycin (PI3K-AKT-mTOR) signaling pathway that regulates genomic stability, cell growth, survival, metabolism, and proliferation [87,88]. PTEN protein levels are regulated by various PTMs, including phosphorylation, ubiquitination, and oxidation [88]. Dempsey et al. (2021) showed that multi-site phosphorylation of PTEN at Ser380/385 and Thr382/383 drives conformational changes in PTEN, inducing the dissociation of phospho-PTEN from the plasma membrane, reduction of catalytic activity and its subsequent ubiquitination [86]. Thus, cell growth control is modulated by ubiquitination-mediated degradation or dysregulated subcellular localization of PTEN [88,89]. USP13, a PTEN deubiquitinating enzyme, can restore PTEN reactivation and stability in tumors [88]. The retinoblastoma protein 1 (RB1) is an important tumor suppressor that acts as a regulator of cell cycle progression via E2F TFs [90,91]. RB1 protein prevents excessive cell growth by suppressing cell proliferation by inhibition of E2F target genes [92]. Cyclin D/CDK4/6-dependent phosphorylation inactivates RB protein, leading to cell cycle progression [92]. The tumor-suppressor cyclin-dependent kinase inhibitor p16 inhibits cyclin D/CDK4/6 activity, enhancing RB activity and suppressing cell growth [92]. The methylation of p16 at Arg138 and the phosphorylation at Ser140 are critical for the modulation of cell proliferation and apoptosis [93]. The Hippo pathway is a key regulator of cell/tissue growth and organ size, and its dysregulation is involved in many cancers [94,95]. The Yes-associated protein (YAP) and transcriptional coactivator with PDZ-binding motif (TAZ) are effectors of the Hippo pathway and are also aberrantly expressed in cancer cells [96]. YAP protein stability, abundance, transcriptional activity, and subcellular localization are modulated by its PTMs, including phosphorylation, ubiquitination, SUMOylation, and acetylation [96]. YAP phosphorylation at Ser127 suppresses YAP activity due to the cytoplasmic retention of YAP that becomes isolated from its nuclear target TFs like transcriptional enhanced associate domain (TEAD) family members, and YAP phosphorylation at Ser381 facilitates its subsequent phosphorylation by CK1 kinase and recognition by E3 ubiquitin ligase, inducing its proteasomal degradation [97]. Conversely, YAP/TAZ accumulation into the nucleus is followed by downstream transcription of genes involved in cell hyperproliferation [97]. The most important PTMs occurring in cancer biomarkers involved in evading growth suppressors are illustrated in Figure 2.

#### 2.1.3. Avoiding Immune Destruction

Cancer progression requires immune evasion, the ability of tumor cells to activate immunosuppressive pathways to avoid an immune response from the host [98]. The programmed cell death protein 1 (PD-1/CD279)/programmed cell death ligand 1 (PD-L1/CD274) signaling pathway is responsible for cancer immune escape [99], suppressing T-cell-mediated anticancer immune responses [100]. Blocking the PD-1/PD-L1 axis is effective in preventing tumor evasion [101]. PD-1 is a transmembrane protein expressed on activated T cells that acts as an important immune checkpoint receptor, which negatively impacts the response to antigens [102]. PD-L1 is a transmembrane protein expressed on the surface of tumor cells that interacts with PD-1 to reduce the proliferation of PD-1-positive cells, inhibit their cytokine secretion, and activate apoptosis of antigen-specific T cells [99].

Ubiquitination and deubiquitination of PD-1 and PD-L1 play key roles in PD-1 regulation and PD-L1 stabilization and dynamics [102]. In addition, glycosylation, phosphorylation, palmitoylation, SUMOylation, and acetylation also play a crucial role in protein stabilization and protein–protein interactions (PPIs) on the PD-1/PD-L1 signaling pathway [102]. PD-1/PD-L1 signaling pathways are negatively regulated by phosphorylation, ubiquitination, ubiquitin-like modification, and methylation, while deubiquitination, glycosylation, palmitoylation, ADP-ribosylation, and deacetylation assure its positive regulation [101]. N-linked glycosylation of PD-L1, found in melanoma, BC, lung, and colon cancers, assures protein stability by a slower turnover rate, PD-1 and PD-L1 interaction, and promotes evasion of T-cell immunity [100,103]. PD-L1 N192, N200, and N219 glycosylation prevents PD-L1 degradation through GSK3-mediated 26S proteasome [104]. In addition, PD-L1 glycosylation impedes antibody recognition of PD-L1 in fixed samples, leading to inaccurate immunohistochemical (IHC) readout in some patient samples and resulting in an inadequate result of anti-PD-1/PD-L1 immunotherapy in clinic [104]. Consequently, deglycosylation of PD-L1 strengthens the signal intensity in PD-L1 IHC assays [100]. Recently, it was demonstrated that ionizing radiation induces translocation of PD-L1 from the membrane into the nucleus after the deglycosylation of PD-L1; thus, PD-L1 deglycosylation accelerates DNA repair in cancer and produces radioresistance [105]. Wang et al. (2021) showed that inhibition of the NEDDylation pathway upregulates PD-L1 expression in gliomas, suppressing cancer-associated immunity [106].

#### 2.1.4. Enabling Replicative Immortality

Telomeres, the repetitive ends of chromosomes, maintain genomic integrity, being major players in enabling replicative immortality, a crucial hallmark of cancer cells [107,108]. Normal human cells undergo a limited number of successive cell divisions and then enter replicative senescence characterized by irreversible cell cycle arrest [109]. Telomere length has been associated with cell proliferation [110]. Over time, telomere shortening occurs through each division of somatic cells and has a dual function in cancer progression [111,112]. The tumor suppressive effect of telomere shortening leads to cell cycle arrest, senescence, and apoptosis, or conversely, loss of telomere can promote genome instability and cancer progression [111]. Telomerase is an enzyme that elongates telomeres, and its abnormal activation occurs in the majority of cancer cells [2,110]. Consequently, cancer cells must reactivate telomerase to extend their telomeres, escape from cellular senescence, and achieve cell immortalization or unlimited proliferative potential [112]. Conversely, in most differentiated human normal cells, telomerase expression is silenced due to the transcriptional repression of human telomerase reverse transcriptase (hTERT), the most important catalytic component of the telomerase enzyme [2,113]. Detectable in up to 90% of human primary cancers [113], TERT is regulated by TFs and PTMs, including phosphorylation, ubiquitination, and SUMOylation, which regulate telomerase integrity and activity, telomere elongation, and tumorigenesis [2]. Sanyal et al. (2020) reported that SUMOylation of hTERT positively regulates telomerase activity, while seSUMOylation inhibits telomerase activity [114]. Moreover, these authors showed that hTERT SUMOylation leads to repression of E-cadherin expression and initiation of the EMT program, promoting invasion and migration in BC cells [114].

#### 2.1.5. Activating Invasion and Metastasis

Invasion of single or clusters of cancer cells into the surrounding stroma is the first step of metastasis, the major cause of death among cancer patients [115,116]. The metastatic cascade, defined as the development of secondary tumors far from the original primary cancer, is also a crucial hallmark of malignancy [117]. Intrinsic characteristics of tumor cells associated with their communication with the tumor microenvironment (TME) contribute to the shaping of this cancer hallmark [117,118]. EMT is an essential step in cancer progression characterized by loss of cell junctions accompanied by downregulation of epithelial cell adhesion molecules, loss of apical-basal polarity, and cytoskeletal remodeling [119].

Circulating tumor cells (CTCs) are released by the primary tumor or metastatic lesions into the blood or the lymphatic system and ensure the intermediate stage of metastasis [118]. Analyzing the expression of the phosphorylation state of circulating tumor cells (CTCs), Payne et al. (2023) identified differences between epithelial, early EMT, and advanced EMT groups of cells [3]. The most important biomarker of CTCs is the epithelial cell adhesion molecule (EpCAM/CD326), a single transmembrane glycoprotein on the cell surface that mediates intercellular adhesion, is overexpressed in the majority of invasive epithelial cancers [120,121]. In BC, EpCAM was found to be hyperglycosylated, whereas deglycosylated EpCAM promoted autophagy and apoptosis, and inhibits proliferation in BC cells through suppressing PI3K/AKT/mTOR pathway [122,123]. Moreover, N-glycosylation mutation of EpCAM inhibits EMT, invasion and metastasis [124]. EpCAM regulates claudins’ dynamic and tight junctions [125]. Claudin-1 (CLN1) is important in tight junction formation, and protein kinase A (PKA) and protein kinase C (PKC) can phosphorylate claudins [126]. French et al., (2009) showed that the subcellular localization of CLN1 can be controlled by phosphorylation that dictates the effects on metastatic ability [126]. Thus, cytoplasmic expression of CLN1 in metastatic melanoma cells was correlated with increased migration, while nuclear claudin-1 expression was found in benign lesions [126].

Cytoskeleton remodeling has a crucial role in cancer cell invasion and migration [127]. Cytoskeletal proteins undergo a broad spectrum of PTMs, which increase the diversity of the cytoskeletal proteome and modulate the function of the cytoskeletal proteins [127]. Thus, phosphorylation of cytoskeletal proteins regulates dynamics of polymerization/filamentation of a plethora of monomers, including actin, myosin, tubulin, keratins, desmins, glial fibrillary acidic protein, peripherin, vimentin, anexins, nestins and others, modulating stability and disassembly of various cytoskeletal polymers, so altered phosphorylation of cytoskeletal proteins was observed in many cancers [128]. MacTaggart and Kashina (2021) summarized the most important PTMs of cytoskeletal proteins [127]. Cancer cell movement involves the formation and elongation of invadopodia, lamellipodia, and filopodia, so the dynamic of actin and actin-binding proteins is essential for cancer cell invasion [129]. Thus, actin (ACT), which represents 1–5% of all non-muscle cell proteins [130], undergoes various PTMs, such as acetylation, arginylation, methylation, oxidation, glutathionylation, S-nitrosylation, nitration, carbonylation, phosphorylation at multiple amino acid residues, ubiquitination, and SUMOylation [127]. Tubulin isoforms undergo acetylation, detyrosination/tyrosination, glutamylation/glycynation, phosphorylation, ubiquitination, SUMOylation, polyamination, and methylation [127]. The intermediate filament vimentin (VIM) protein is upregulated during EMT to enhance the invasiveness of carcinoma cells [131]. Thus, VIM is a biomarker expressed in metastatic cancers, where it is required for tumor progression and metastasis [132]. VIM protein undergoes phosphorylation, methylation, glycosylation, acetylation, citrullination, ADP-ribosylation, S-nitrosylation, S-glutathionylation, SUMOylation, and ubiquitination [127,133]. VIM phosphorylation is critical for its structure and function [131]. Thus, Kuburich et al. (2023) demonstrated that stabilizing VIM phosphorylation inhibits stem-like characteristics and metastasis of hybrid epithelial/mesenchymal carcinoma cells [131]. It was also demonstrated that VIM controls collagen deposition and extracellular matrix structure (ECM) via posttranscriptional regulation of ECM-related genes [133]. In gastric cancer cell lines, deubiquitination stabilizes VIM to contribute to malignancy [134]. VIM is frequently citrullinated in cells during EMT of metastasizing epithelial tumor cells [8]. Loss of keratins 8 and 18 (K8/18) is also a hallmark of EMT, increasing collective cell migration [135]. K8 and K18 also undergo phosphorylation, methylation, glycosylation, acetylation, SUMOylation, and ubiquitination [127]. In addition, PTMs of EMT-inducing transcription factors (EMT-TFs), such as phosphorylation, ubiquitination, acetylation, methylation, glycosylation, SUMOylation of SNAIL, SLUG, ZEB1/2, and TWIST1 induce their activation or destabilization, nuclear/cytoplasmic shuttle, promoting EMT or, contrarily, mediating their destruction via proteasomal degradation [136]. On the other hand, succinylation of S100A10, a member of S100 calcium-binding cytosolic proteins involved in invasion and migration of tumor cells, increases human gastric cancer invasion, by suppression of its ubiquitination and subsequent proteasomal degradation [137]. Hypoxia-inducible factor 1α (HIF1α) activation is also a factor that can induce EMT in tumor cells [138]. HIF1α activity is modulated by multiple PTMs that affect its stability and transcriptional activity, including acetylation, ubiquitination, and propionylation [46].

**Figure 3:**
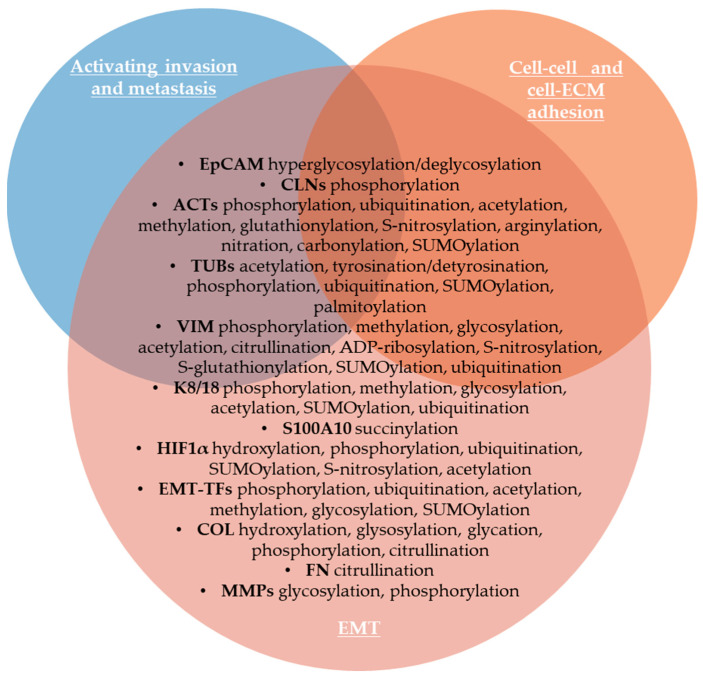
PTMs of key biomarkers of cancer involved in activating invasion and metastasis. ACTs—actin proteins; CLNs—claudins; COL—collagen molecules; EMT-TFs-epithelial-to-mesenchymal transition-transcription factors; EpCAM—epithelial cell adhesion molecule; HIF1α—hypoxia-inducible factor 1-alpha; FN—fibronectin; K8/18—cytokeratin 8/18; MMPs—matrix metalloproteinases; S100A10—S100 calcium-binding protein A10; TUBs—tubulins; VIM—vimentin.

The extracellular matrix (ECM), mainly consisting of proteoglycans, glycoproteins, and matrisome proteins, also plays an important role in tumor development [139]. Evidence suggests that EMT can be induced in cancer cells by adhesion to type I collagen [140]. Collagen family members, the most abundant proteins in ECM, undergo extensive PTMs, affecting receptor binding, cell migration, and tumor stiffness [141]. Borst et al. (2024) showed that collagen PTMs include proline and lysine hydroxylation that takes place in the endoplasmic reticulum (ER), glycosylation and glycation occur in the ER and Golgi apparatus, and collagen can also be modified by phosphorylation and citrullination [141]. Thus, targeting intracellular PTMs can inhibit collagen secretion and deposition, remodeling ECM to improve immune cell migration [141]. Citrullination of other matrisome proteins, including fibrinogen and fibronectin (FN), as well as matrix metalloproteinases (MMPs), has been reported in cancer [57]. MMPs, their substrates, and ECM-binding partners are modified by PTMs, including glycosylation and phosphorylation [11]. The most important PTMs of cancer biomarkers involved in EMT, as well as in activating invasion and metastasis, are listed in Figure 3.

#### 2.1.6. Inducing or Accessing Vasculature

The previous cancer hallmark, “inducing angiogenesis”, has been more recently replaced by ”inducing or accessing vasculature” due to the discovery of non-angiogenic tumors able to exploit normal vessels [142]. Sonic hedgehog (SHH) and its signaling pathway promote neovascularization by capillary morphogenesis and induce endothelial cell migration, contributing to the development and progression of many cancers [143,144]. Zinc-finger protein glioma-associated oncogene 1 (GLI1) is a transcriptional effector of hedgehog signaling, and its aberrant expression was linked to many hallmarks of cancer, including angiogenesis, cell proliferation, survival, metastasis, metabolic reprogramming, and chemotherapeutic resistance [145]. Recently, Lei et al. (2024) showed that GLI1 overexpression enhanced the endothelial cells and pericytes’ motility required for angiogenesis in non-small cell lung cancer [146]. GLI1 proteins are modified by PTMs, including phosphorylation, ubiquitination, SUMOylation, acetylation, and O-GlcNAcylation, which regulate its intracellular trafficking and transcriptional activity of the SHH signaling pathway [147]. GLI1 translocates from the cytoplasm to the nucleus, targeting the transcription of various downstream genes [145,146]. The nuclear GLI1 is acetylated by p300 or CBP at K518, the AcGLI1 being temporarily inactive, while deacetylation allows GLI1 to initiate transcription of target genes [148].

#### 2.1.7. Resisting Cell Death

Apoptosis is a major form of programmed cell death or cell suicide, and evasion of apoptosis or aberrant apoptosis is a hallmark of cancer that results in uncontrolled cell proliferation in tumors [149,150,151]. The extrinsic or death receptor-mediated apoptotic pathway and the intrinsic or mitochondrial-mediated pathway are two distinct core branches of apoptosis [152]. Caspases are crucial players in apoptosis [153].

The extrinsic pathway is initiated by cell surface death receptors like tumor necrosis factor receptor (TNFR) superfamily, FasR (CD95), and TRAIL R1/2 (also called death receptor DR4/5), which bind with tumor necrosis factor α (TNFα), Fas ligand (FasL/CD95L), and TNF-related apoptosis-inducing ligand (TRAIL/APO2) [154,155]. Death receptor activation leads to the activation of pro-caspases 8 and 10 into active caspases 8 and 10 [154]. The intrinsic apoptosis pathway is regulated by the B-cell lymphoma 2 (Bcl-2) family proteins [155]. Cancer cells prevent apoptosis through modulation of Bcl-2 proteins, via upregulation of anti-apoptotic and pro-survival proteins, such as BCL-2, BCL-XL, and MCL1, or/and downregulation of pro-apoptotic proteins, such as BAX, BAK, and BOK [150]. Internal and external stresses lead to the release of cytochrome *c* from mitochondria during the intrinsic or stress-mediated apoptotic pathway that activates pro-caspase 9 in caspase 9 that activates caspase 3 [154].

Both apoptotic pathways are strongly regulated by PTMs, mainly by ubiquitination [156]. Phosphorylation is also a key process in the TNFR1-mediated signaling pathway [157]. Both phosphorylation and ubiquitination highly regulate caspase 8 activity [158]. Thus, caspase 8 is phosphorylated at Tyr380 by Src kinase upon EGF receptor stimulation, which suppresses apoptosis and participates in tumor progression [153], while pro-caspase 8 can be phosphorylated at Ser387 by pERK1/2 in a cell cycle specific manner, also inhibiting caspase 8 induced apoptosis in ovarian and BC cell lines [154]. Caspase 8 is modified with ubiquitin, which mainly inhibits its pro-apoptotic functions [156].

**Figure 4:**
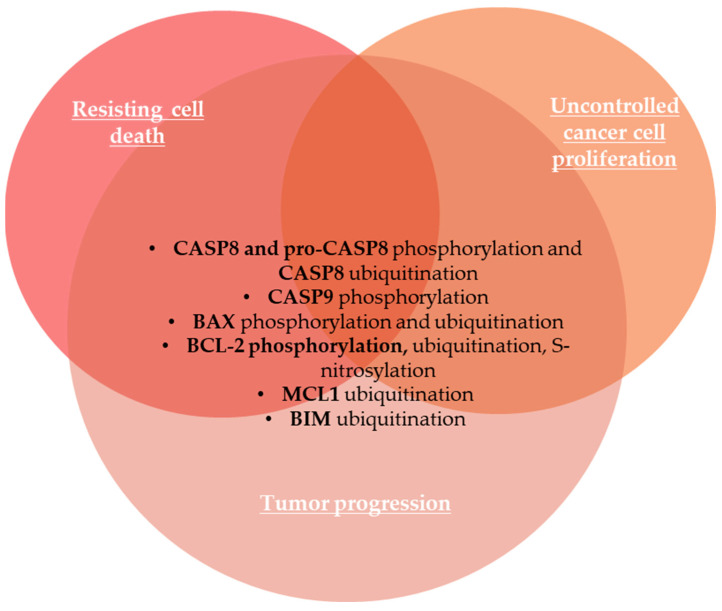
PTMs of key biomarkers of cancer involved in resisting cell death in association with uncontrolled cancer cell proliferation and tumor progression. BAX—BCL-2-like protein 4; BCL-2—B-cell lymphoma2/leukemia protein; BIM—BCL-2-like protein 11/BCL-2 interacting mediator of cell death; CASP8—caspase 8; CASP9—caspase 9; MCL1—myeloid leukemia 1 protein.

Pro-apoptotic BAX protein is mainly distributed in the cytoplasm in the inactive form [152]. Upon apoptotic stimuli, BAX proteins are activated, undergo a conformational change, and then are translocated to the mitochondria’s outer membrane [152]. BAX activation induces mitochondrial outer membrane permeabilization, followed by the release of cytochrome *c* and other pro-apoptotic factors, leading to cancer cell death [152,159]. In the intrinsic apoptotic pathway, ubiquitination targets both anti-apoptotic and pro-apoptotic proteins [156]. For example, PTMs of BAX, including phosphorylation and ubiquitination, play key roles in the regulation and stabilization of BAX proteins [160]. Thus, phosphorylation of BAX at Ser184 by protein kinase B (PKB), a serine/threonine kinase also known as AKT, converts pro-apoptotic BAX protein into an anti-apoptotic protein, thus inhibiting apoptosis in cancer cell lines [161]. Moreover, BAX can undergo degradation through the ubiquitin–proteasome pathway [152]. IBRDC1, an IBR-type RING-finger E3 ubiquitin ligase, regulates BAX levels through increased BAX ubiquitination, degradation, and downregulation, while IBRDC1 downregulation leads to accumulation of activated BAX and apoptosis [162]. The oncogenic beclin-2 (BCL-2) is a key anti-apoptotic protein of the mitochondrial pathway, so BCL-2 PTM, such as ubiquitination and proteasomal degradation, are known as regulators of BCL-2 function [163]. BCL-2 undergoes S-nitrosylation by endogenous nitric oxide (NO) that inhibits its ubiquitin-proteasomal degradation, thus preventing BCL-2 downregulation and apoptosis [163]. Phosphorylated BCL-2 at Ser70 exhibits enhanced binding to BIM and BAK pro-apoptotic proteins during mitosis, leading to increased sequestration of BAK and BIM [164]. The chemotherapeutic drug cisplatin initiates an apoptotic pathway by phosphorylation of anti-apoptotic BCL-XL by cyclin-dependent kinase 2 (CDK2) [165]. Thus, phosphorylated BCL-XL translocates to the outer mitochondrial membrane, forms pores, and increases membrane permeabilization, cytochrome c release, and caspase activation, leading to cell death [165]. Ubiquitination targets both anti-apoptotic proteins, like MCL1, as well as pro-apoptotic proteins like BIM [156]. Caspase 9, an important player in the initiation of the intrinsic apoptosis pathway, is frequently phosphorylated at Thr125 by the mitogen-activated protein kinase (MAPK) pathway, leading to inhibition of caspase 9 activation and caspase 9-mediated apoptosis in gastric carcinoma [166]. PTMs of proteins involved in resisting cell death are summarized in Figure 4.

#### 2.1.8. Deregulating Cellular Metabolism

Cancer can be characterized as a metabolic disease and metabolic reprogramming is considered to be the most important emerging hallmark of tumor cells [20,167]. Malignant cells emphasize increased aerobic glycolysis, glutaminolysis, and de novo lipogenesis to assure their survival and increased proliferation [168,169]. Thus, malignant cells manage the ability to acquire necessary nutrients from a nutrient-poor environment and develop reprogrammed glucose metabolism that is essential for cell growth and survival [170,171]. The most studied altered metabolism in cancer is aerobic glycolysis or the Warburg metabolism/effect, which refers to the switch from the normal respiratory pathway to aerobic glycolysis, resulting in a high amount of lactate in the cytosol and ECM [167,172].

Tyrosine phosphorylation influences the activity of some metabolic enzymes, being essential for reprogramming the energy metabolism in cancer cells [173], as well as ubiquitination and deubiquitination [169]. The rate-limiting glycolytic hexokinase (HK) isoenzymes, including HK1 with many isoforms [174], HK2, HK3, HK4 (also named glucokinase (GK)), and hexokinase domain-containing protein 1 (HKDC1), phosphorylate glucose to produce glucose-6-phosphate [170] that then initiate major pathways of glucose utilization including glycolysis, pentose phosphate pathway (PPP) and oxidative phosphorylation (OXPHOS) [175]. Hexokinase activity is regulated through protein expression as well as PTMs of HKs [170]. Thus, HK2 phosphorylation by AKT2 at the threonine 473 residue (T473) promotes its binding to mitochondria, which increases HK2 catalytic activity and enhances glycolysis, promoting tumorigenesis in colon cancer cells and lung metastasis in nude mice through upregulation of nuclear factor-kB (NF-kB), hypoxia-inducible factor 1α (HIF1α), matrix metalloproteinase 2 (MMP2), and matrix metalloproteinase 9 (MMP9) upregulation [176]. Both HK1 and HK2 phosphorylation, especially HK1 phosphorylation at tyrosine 732 (Tyr732/Y732) by c-Src, increases their catalytic activities and enhances glycolysis in various cancer cell lines and tumor types so that the phosphorylation level of HK1-Y732 may be used as a novel biomarker to predict metastasis risk of primary tumors [177]. HK2 shuttles from the cytoplasm to the mitochondria in response to environmental and metabolic stress, so HK2 ubiquitination by HectH9 E3 ligase at lysine K63 residue regulates its mitochondrial localization, regulating cancer stem-cell (CSC) expansion, promoting glycolysis, inhibiting apoptosis, and enhancing CSC-associated drug resistance [175]. Consequently, K63-linked ubiquitination activates kinases and TFs, targets transmembrane proteins for lysosomal degradation, and modulates protein trafficking [175]. Guo et al. (2023) also summarized other PTMs of hexokinases, such as SUMOylation of HK2 in prostate cancer cells, HK1 palmitoylation in hepatic stellate cells, and S-nitrosylation of HK4 [170]. Pyruvate kinase plays an essential role in regulating cell metabolism and contributes to tumorigenesis [167].

Pyruvate kinase M2 (PKM2) has an upregulated expression in most cancer cells, where it tends to exist as a dimer and drives the Warburg effect for tumor progression [167,178]. Zhou et al. (2024) demonstrated that PKM2 phosphorylation at Ser333 antagonizes O-GlcNAcylation, promotes its tetramer formation, decreasing its nuclear localization, and thus inhibiting c-Myc expression [178]. Consequently, PKM2 phosphorylation and tetramer formation attenuate the Warburg effect in cancer cells by downregulation of glucose consumption and lactate production, whereas phospho-PKM2 deficiency promotes tumor growth in vivo [167,178].

**Figure 5:**
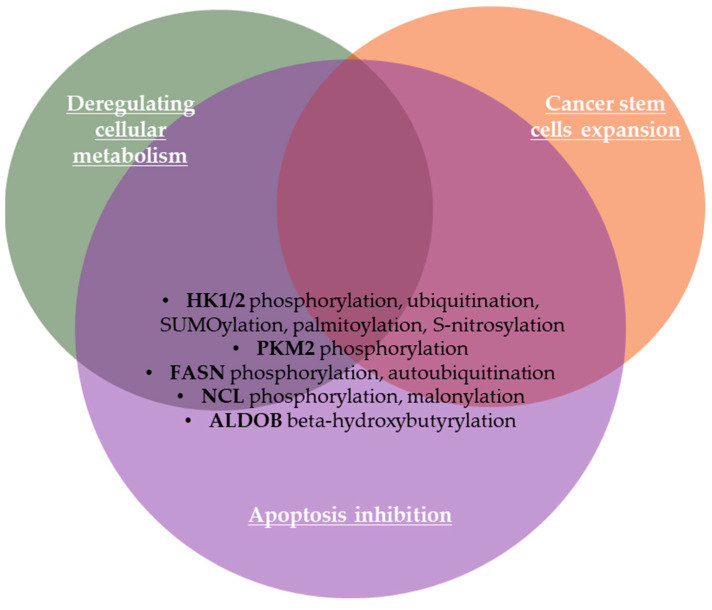
PTMs of protein biomarkers of cancer involved in deregulating cancer cell metabolism in association with cancer stem-cell expansion and apoptosis inhibition. ALDOB—aldolase B; FASN—fatty acid synthase; HK1/2—hexokinases 1/2; NCL-nucleolin; PKM2—pyruvate kinase M2.

The fatty acid synthase (FASN), an enzyme involved in de novo fatty acid synthesis, is overexpressed in many cancers [179], including colorectal cancer [168]. FASN promotes cell proliferation through membrane biosynthesis, energy storage, generation of signal mediators, resistance to apoptosis, cell invasion, immune escape, metastasis, and angiogenesis [168,179]. Jin et al. (2010) showed that FASN phosphorylation by human epidermal growth factor receptor 2 (HER2) plays a key role in HER2-overexpressing BC progression [180]. FASN cytoplasmic degradation can be prevented by autoubiquitination and degradation of enhanced F-box and WD repeat domain-containing 7β (FBXW7β), a cytoplasmic isoform of FBXW7 component of ubiquitin E3 ligase complex involved in FBXW7β/GSK3β-mediated FASN ubiquitination, resulting in FASN-mediated lipogenesis and promotion of colorectal cancer growth [168]. Nucleolin (NCL) is a multifunctional protein distributed in the nucleoli, nucleoplasm, cytoplasm, and plasma membrane. NCL is also overexpressed in many cancers, including BC, gastric cancer, leukemia, and glioblastoma, due to its roles in regulating proliferation, survival, and metastasis of cancer cells through its involvement in ribosome biogenesis, mRNA translation, and chromatin remodeling [20,181]. Recently, Sun et al. (2024) showed that PTMs, in particular phosphorylation, play an important role in the subcellular localization and function of NCL [20]. These authors also found that NCL is highly malonylated in hepatocellular carcinoma [20]. Thus, malonylated NCL translocates from nucleolus to nucleoplasm, binds to AKT mRNA, and facilitates AKT translation, promoting cell proliferation [20]. These findings suggest malonylation as a novel mechanism controlling cancer cell proliferation based on the potential communication between lipid metabolism and protein function regulated by PTMs [20]. Succinylation also has a key relationship with energy metabolism, participating in fatty acid synthesis, amino acid degradation, electron chain transmission, ketone body formation, the TCA cycle, and other metabolic processes [22]. Cancer cell metabolism can be reshaped by aldolase B (ALDOB) β-hydroxybutyrylation at lysine 108 (Lys108) induced by a ketogenic diet, inhibiting its enzymatic activity [23]. These authors demonstrated that attenuation of ALDOB activity inhibits mammalian/mechanistic target of rapamycin (mTOR) signaling and glycolysis, suppressing hepatocellular carcinoma cell proliferation [23]. PTMs of proteins involved in deregulating cellular metabolism in cancer are summarized in Figure 5.

### 2.2. PTMs and Cancer Enabling Characteristics

#### 2.2.1. Genome Instability and Mutation

The integrity of the genome is controlled by DNA damage signaling and DNA damage repair (DDR) [182]. Genomic instability (GI) is a hallmark of most cancer cells during cell division due to mutations in DNA repair genes or mitotic checkpoint genes, which predispose cells to malignant transformation [183]. GI is a direct consequence of the inactivation of the tumor-suppressor protein p53, as well as the p53 pathway, which is inactive in the majority of cancer cells [184]. p53, encoded by the *TP53* gene, is known as the ”guardian of the genome” for its role in the preservation of genomic stability [185]. p53 undergoes multiple PTMs that modulate its diverse functions in specific biological contexts [186]. Wen and Wang (2021) highlighted the potential of ”PTMomic” studies of p53 for cancer therapy [186]. In response to DNA damage, p53 undergoes acetylation and deacetylation with consequences on sequence-specific DNA-binding activities [186]. Crotonylation, β-hydroxybutyrylation, hydroxylation, and methylation have also been cited as PTMs that occur in p53 [186]. Thus, under cellular stress, p53 undergoes phosphorylation via a series of kinases, including ataxia telangiectasia-mutated serine/threonine kinase (ATM), ATM and RAD3-related serine/threonine kinase (ATR), and checkpoint kinase 1/2 (CHK1/CHK2) [186], phosphorylation stabilizing p53 by disrupting its interactions with mouse double minute 2 (MDM2) oncoprotein, an E3-ubiquitin ligase that triggers p53 degradation through ubiquitin–proteasome-dependent pathway [187]. Consequently, p53 is translocated into the nucleus, where it contributes to transcription of target genes. Moreover, in cells where the p53 was activated by stress, p53 polyubiquitination is switched to p53 monoubiquitination, maintaining its nuclear localization and DNA-binding activity [14]. p53 is also modified by β-hydroxybutyrylation at lysine K120/319/370 residues, which attenuates its activity and explains the link between ketone bodies and cancer [188].

The germline mutations in breast cancer susceptibility genes *BRCA1* and *BRCA2* have been linked to genomic instability [182]. In addition, the function of breast cancer tumor-suppressor 1 (BRCA1) protein is regulated by phosphorylation and acetylation [189]. BRCA1 is a nuclear phosphoprotein that plays several roles in genome stability, including checkpoint promotion, DDR, replication fork stability, and DNA double-strand break repair [190]. BRCA1 regulates transcription loss, leading to genome instability and malignant transformation [191]. Thus, CHKs phosphorylate specific residues of BRCA1 under conditions of DNA damage, modulating the response of cells to various stresses [191]. BRCA1 is part of an obligate multifunctional heterodimer with BRCA1-associated RING domain 1 (BARD1), acting together as a tumor-suppressor E3 ubiquitin ligase involved in repairing double-strand DNA breaks through homologous recombination, maintaining genome stability [190,192]. Lahusen et al. (2018) emphasized the role of BRCA1 acetylation at K830 in the activation of BRCA1 function at the intra-S checkpoint after DNA damage [189]. GATA binding protein 3 (GATA3) is a nuclear zinc-finger TF that functions downstream of BRCA1 to promote DDR and suppress dedifferentiation in BC, acting as a tumor suppressor [193]. PTMs of GATA3 are crucial for its function [194]. The progestin-activated progesterone receptor (PR) reduces GATA3 expression by GATA3 phosphorylation at Ser308, followed by its proteasome-mediated degradation [195]. Thus, PR activation downregulates GATA3 by transcriptional repression and increases protein turnover, promoting BC growth [195].

#### 2.2.2. Tumor-Promoting Inflammation

**Figure 6:**
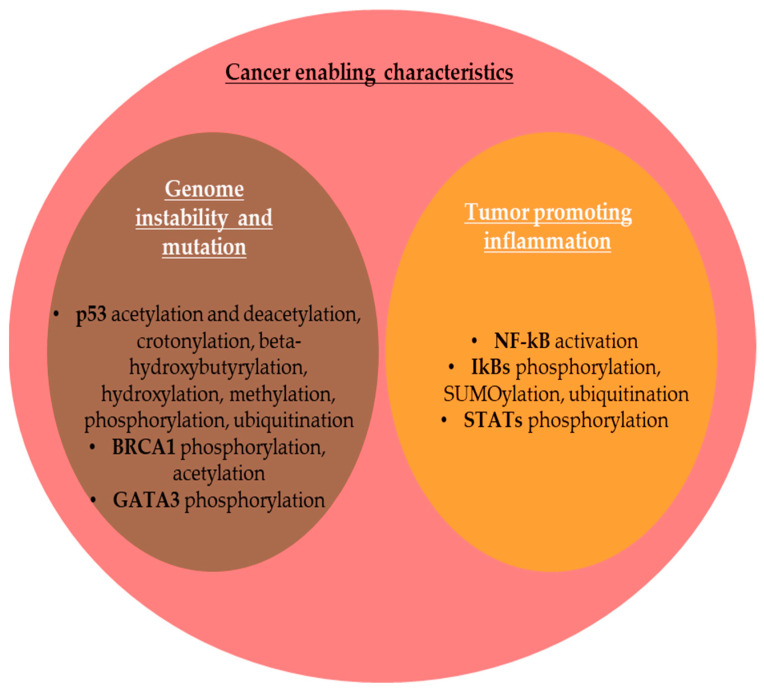
PTMs of protein biomarkers involved in cancer enabling characteristics. BRCA1—breast cancer type 1 susceptibility protein; IkBs—inhibitors of kappa B; NF-kB—nuclear factor kappa B; STAT—signal transducer and activator of transcription.

Inflammation either promotes or suppresses tumor progression [196]. Thus, chronic inflammation promotes tumor progression and treatment resistance, while induction of acute inflammation leads to anti-tumor immune responses [196]. The nuclear factor of a k-light chain of enhancer-activated B cells (NF-kB) is a pro-inflammatory family of TFs that activate the transcription of key genes that connect tumorigenesis and inflammation [197,198]. In the cytoplasm, NF-kB binds to the inhibitors of kappa B (IkBs) responsible for maintaining NF-kB in an inactive state [199,200]. The activation of the NF-kB pathway involves phosphorylation of IkBs [200]. SUMOylation of IkBa was also recognized as a regulator mechanism for the NF-kB signaling pathway [60]. As a consequence of the phosphorylation and ubiquitination of IkB proteins, NF-kB is dissociated and translocated into the nucleus to bind with DNA for inducing the transcription of immune and inflammatory cytokines [197,198]. The Janus kinase (JAK)/signal transducer and activator of transcription (STAT) signaling is a key pathway involved in tumorigenesis by mediating the cellular response to inflammation [201]. STAT proteins are inactive in the cytoplasm, but phosphorylation leads to conformational changes in the STATs, which migrate into the nucleus and regulate the expression of target genes upon activation by several cytokines, such as interleukin 6 (IL-6), interleukin 10 (IL-10), and interleukin 11 (IL-11) [202,203]. JAKs phosphorylate mainly STAT1 and STAT3, activating their nuclear translocation [203,204,205]. PTMs of cancer biomarkers involved in tumor-enabling characteristics are summarized in Figure 6.

### 2.3. PTMs Involved in Additional Emerging Hallmarks and Enabling Characteristics of Cancer

**Figure 7:**
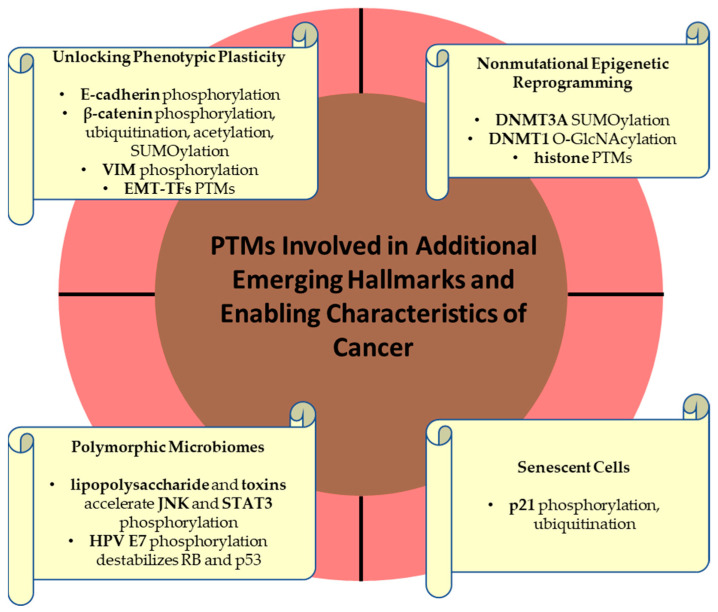
PTMs of proteins involved in additional emerging hallmarks and enabling characteristics of tumors. DNMT1—DNA (cytosine-5)-methyltransferase 1; DNMT3A—DNA (cytosine-5)-methyltransferase 3A; EMT-TFs—epithelial-to-mesenchymal transition-related transcription factors; HPV E7—Human Papilloma Virus E7 oncoprotein; JNK—c-Jun N-terminal kinase; p-21—cyclin-dependent kinase inhibitor 1; STAT3—signal transducer and activator of transcription 3; VIM-vimentin.

In 2022, Hanahan incorporated four additional proposed emerging hallmarks and enabling characteristics involving unlocking phenotypic plasticity, nonmutational epigenetic reprogramming, polymorphic microbiomes, and senescent cells. PTMs of protein also orchestrate these characteristics of cancer (Figure 7).

#### 2.3.1. Unlocking Phenotypic Plasticity

Cell plasticity refers to the ability of cells to be reprogrammed, enabling them to acquire novel molecular, phenotypic, and functional traits that allow for tumor initiation, progression, metastasis, and therapy resistance [206,207]. The epithelial-to-mesenchymal transition (EMT) is a reversible form of cellular plasticity by which cancer cells undergo molecular, phenotypic plasticity, and behavioral transition from an epithelial to a mesenchymal state, increasing their migratory and invasive features [208]. Dysregulation of cadherin-catenin complexes, crucial components of the adherens junctions, was linked to cancer development [209]. The hallmark of EMT is the upregulation of neural cadherin (N-cadherin) followed by loss of functional epithelial cadherin (E-cadherin), a switch orchestrated by various signaling pathways and transcription factors (TFs) [210]. Thus, PTMs of cadhesome proteins, including phosphorylation at serine, threonine, and tyrosine residues, play a key role in the regulation of adherens junctions [210]. In addition, activation of EMT-inducing transcription factors (EMT-TFs), such as zinc-finger protein SNAI1 (SNAIL), zinc-finger protein SNAI2 (SLUG), zinc-finger E-box binding homeobox 1/2 (ZEB1/ZEB2), and twist-related protein 1/2 (TWIST1/2), leads to transcription of genes that are involved in mesenchymal programs and inhibition of genes that drive the epithelial differentiation [211]. These EMT-TFs mediate the transcriptional repression of E-cadherin and upregulation of vimentin [132,212] and undergo PTMs to regulate metastasis phosphorylation, mainly affecting the flexibility and reversibility of EMT [213]. The silencing of E-cadherin expression, a tumor-suppressor protein from epithelial adherens junctions, has been associated with the destruction of intercellular adhesion mechanisms and EMT, significantly occurring during tumor metastasis [214]. Phosphorylation of E-cadherin at Thr790 by protein kinase C (PKC) suppresses the function of E-cadherin by diminishing its interaction with β-catenin in cervical cancer tissues [215]. Moreover, β-catenin itself can emphasize various subcellular localizations and functions orchestrated by several PTMs. Thus, β-catenin is regulated by WNT signaling, so in the absence of extracellular WNT signals, the expression of β-catenin is kept at a low level in the cytoplasm through the ubiquitin–proteasome system [216]. For this purpose, β-catenin is phosphorylated by casein kinase 1 (CK1) and then by glycogen synthase kinase 3 beta (GSK3β), leading to its subsequent ubiquitination and proteasomal degradation [216]. The activation of WNT signaling pathway, conversely, leads to β-catenin stabilization through inhibition of phosphorylation or inactivation of ubiquitination, followed by its accumulation in the cytoplasm and successive translocation into the nucleus, where it initiates the transcription of WNT target genes, such as *c-Myc*, *Jun*, and *cyclinD-1* oncogenes [216]. Evidence suggests that protein lysine acetylation is crucial for WNT/β-catenin signaling activation [217]. You et al., (2022) concluded that β-catenin acetylation improves its stability by inhibiting the ubiquitin-mediated degradation and induces its nuclear translocation to enhance the transcription of WNT target genes [217]. In addition, β-catenin SUMOylation in melanoma cell lines stabilizes β-catenin and inhibits its degradation by the proteasomal system [218]. The intermediate filament VIM protein is highly overexpressed in metastatic cancers, where it facilitates the formation and elongation of the invadopodia that facilitate cancer cell invasion and migration [132]. The phosphorylation of VIM at Ser39 enhances the ability of VIM to induce motility and invasion, also protecting VIM from caspase-induced proteolysis [219]. SNAIL, SLUG and TWIST1 are major EMT-TFs that undergo nuclear-cytoplasmic shuttling [220] and activate genes associated with the mesenchymal-migratory phenotype of cancer cells [221]. EMT-TFs degradation is initiated by specific phosphorylation, followed by subsequent ubiquitination and destruction in the ubiquitin–proteasome system [211]. Conversely, deubiquitination stabilize TFs, accelerating the EMT program [221]. SUMOylation of SNAIL in a prostate carcinoma cell line is essential for the EMT-activating function of SNAIL [211,222]. SLUG acetylation increases its stability in BC cells [223] as well as SNAIL acetylation, which promotes invasion and metastasis in lung cancer cells [224]. SUMOylation of TWIST2 through ligase activity of ZNF451 stabilizes TWIST2 and promotes EMT [225].

#### 2.3.2. Nonmutational Epigenetic Reprogramming

Dysregulation of the epigenetic program is a hallmark of cancer [226]. Reversible epigenetics modifications, including DNA hypo- or/and hypermethylation [227], PTMs of proteins including histone and non-histone protein modifications [228], and noncoding RNAs (ncRNAs) affect gene expression in cancer cells by alteration of chromatin and DNA accessibility without changes on DNA sequences [19,229]. Epigenetic changes lead to activation of oncogenes and silencing of tumor-suppressor genes, thereby encouraging tumorigenesis and cancer progression [226].

DNA methylation is catalyzed by DNMT1 as well as de novo DNMT3A and DNMR3B methyltransferases aberrantly expressed in cancer [227,230]. Regulation of chromatin biology and gene transcription is modulated by DNMT3A binding with many proteins [231]. DNMT3A enzyme is modified by SUMO1, and SUMOylation disrupts DNMT3A-histone deacetylases (HDAC) interaction, thus changing histone acetylation pattern and eliminating the ability of DNMT3A to repress transcription [231,232]. Thus, SUMOylation was recognized as an important mechanism to regulate gene transcription [232]. In addition, O-GlcNAcylation impacts DNA methylation through modulation of DNMT1 activity and ten-eleven translocation 1/2/3 (TET1/2/3) stability and DNA binding [233].

Histone proteins play a central role in gene regulation [56]. Aberrant histone modification affects genome stability and disrupts gene expression, leading to cancer and other diseases [228]. Histone PTMs are covalent changes in histone tails, including H2A, H2B, H3, and H4 [228]. PTMs of histones, as well as other chromosomal proteins, regulate chromatin conformation and gene transcription [234]. Histone PTMs, including methylation, acetylation, phosphorylation, ubiquitination, SUMOylation, NEDDylation [235], acylation [236], crotonylation [237], lysine lactylation [238], borylation and propionylation [239], succinylation [240], citrullination [55,56], malonylation, glutarylation, hydroxybutyrylation, benzoylation, O-GlcNAcylation [233], biotinylation [241,242], tyrosine hydroxylation, ADP-ribosylation [243], N-formylation at multiple lysyl residues [234], proline isomerization, and can contribute to chromatin remodeling, nucleosome dynamics, and transcription of key oncogenes, tumor-suppressor genes, and TFs [6,228]. Abnormal histone methylation or demethylation in the corresponding cancer-related genes contributes to carcinogenesis, proliferation, metabolic rewiring, EMT, invasion, and migration in digestive cancers, including gastric, liver cancer, pancreatic cancer, and colorectal cancer [244]. Histone acetylation and deacetylation are essential for genome integrity, DNA replication, DDR, and RNA transcription [228,245]. Li et al. (2014) showed that H2A NEDDylation antagonizes its ubiquitination [235]. Thus, NEDD8 negatively regulates DDR by suppressing the ubiquitination of H1A, also blocking the recruitment of damage-response protein BRCA1 [235]. In addition, aberrant histone phosphorylation can also mediate tumor development and metastasis [228]. Histone formylation is involved in nucleosome organization and DNA binding [234]. Biotinylation of histones plays a role in cell proliferation, gene silencing, and DDR [242]. Kebede et al. (2017) emphasized that histone acetylation, butyrylation, and propionylation act in a combinatorial mode to promote elevated transcription and to associate cellular metabolism with the structure and function of chromatin [239]. In cancerous tissue, histone lysine lactylation (KIa) has been associated with metabolic reprogramming, mainly glucose and glutamine metabolism, thus contributing to tumorigenesis, immune evasion, and angiogenesis [238]. The poly(ADP-ribose) polymerase 1 (PARP1) is activated by DNA damage and is able to recruit other proteins to repair DNA single-strand breaks, and PARP inhibitors (PARPi) are an important class of anticancer therapies that target tumors with BRCA1/2 mutations, such as ovarian, BC, lung and pancreatic cancer [246]. Histone ADP-ribosylation is essential for PARP1 release from DNA lesions, thus contributing to resistance to PARP inhibitors [243]. Histone citrullination also plays functions in tumors [56].

#### 2.3.3. Polymorphic Microbiomes

Recently, updated definitions for the term microbiome have been proposed. Thus, the term microbiome is actually used to define a complex microbial ecosystem [247], containing both the community of microorganisms, known as microbiota, which includes bacteria, archaea, fungi, protozoa, and viruses [248], and their milieu, including microbial structural elements, such as proteins/peptides, lipids, polysaccharides and nucleic acids, microbial metabolites, including signaling molecules, toxins and other organic and inorganic molecules, as well as other microenvironmental conditions [249]. The most studied in association with cancer is the gut microbiome, but other microbiomes from different tissues and organs, such as lung, genitourinary, skin, and breast, as well as the intratumoral microbiome, emphasized an increased interest in cancer research [247].

Polymorphic microbiomes are involved in tumorigenesis and oncotherapy response [247,250]. Recently, dysbiotic microbiota was recognized as an important emerging hallmark of cancer [58,251]. Small molecule metabolites produced by tumor microbiota diffuse into tissues and cells, remodeling signaling pathways of cancer and cancer-associated cells [252]. For example, it is known that gut microbiota may promote colon tumor formation [253] and can be considered to be a new prognostic biomarker and a putative therapeutic target [254]. Gut bacteria emphasize estrogen metabolizing properties and it was also suggested that breast microbiota could originate in the gut microbiota via bacterial spreading through mesenteric lymph nodes [250]. Thus, Li et al. (2012) demonstrated that gut microbiome, through lipopolysaccharide, a major cell-wall component of Gram-negative bacteria that promotes colon cancer metastasis [255], accelerates tumor growth through phosphorylation of c-Jun/Janus kinase (JNK) and STAT3 [256]. STAT3 regulates the transcriptional activation of several anti-apoptotic and pro-proliferative genes, such as *BCL-2*, so attenuation of STAT3 phosphorylation can promote apoptosis in human osteosarcoma [257]. The human enterotoxigenic *Bacteroides fragilis* secretes *a* specific toxin that enhances STAT3 phosphorylation and nuclear translocation [253]. *Helicobacter pylori*, a class 1 human carcinogen considered to be one of the most important risk factors in gastric cancer, produces cytotoxin-associated gene A (CAGA) [258], which is first translocated into the epithelial cells and then is tyrosine phosphorylated by Src and tyrosine-protein kinase ABL, resulting in significant elongation and motility of infected cells [259]. In addition, Piao et al., (2020) demonstrated that *H. pylori* infection induces STAT3 phosphorylation at Ser727, localization of pSTAT3 mainly in mitochondria, and autophagy in human gastric epithelial cells [260]. Mitochondrial localized pSTAT3 controls the activity of the electron transport chain and also promotes BC growth [261]. Viral genomes can be integrated into the host cellular genome [262,263]. Consequently, in human papillomavirus (HPV)-induced cervical cancer, two major viral oncogenes/oncoproteins, HPV E6 and E7, are able to destabilize tumor suppressors p53 and retinoblastoma protein (RB), inducing all hallmarks of cancer, mainly blocking apoptosis and initiating cell division, which result in tumor formation [263,264]. Recently, Malone et al., (2024) showed that the protein E7 from high-risk HPV is serine phosphorylated by the casein kinase 2 (CK2) to a higher degree compared to low-risk HPV, this phosphorylation event increasing the binding activity for tumor-suppressor pRB that leads to an increased ability of E7 to enhance cellular proliferation and reduce senescence [264].

#### 2.3.4. Senescent Cells

Senescence is a hallmark of cancer [265]. Cellular senescence acts as a tumor-suppressor mechanism by preventing the growth of damaged cells, but it can also play, under certain conditions, a tumor-promoting role by building an inflammatory microenvironment [265,266,267]. This cellular state involves a permanent cell cycle arrest, altered metabolism, a senescence-associated secretory phenotype (SASP), and molecular damage [268]. Cancer cells emphasize senescence escape by which cancer cells regain their proliferative potential [269]. p53-p16 and p21-RB, mTOR, MAPK, and PI3K are major pathways involved in senescence [265,269]. p21 is a cyclin-dependent kinase inhibitor, which plays an important role in senescence [270]. In many human tumor cells, proteins involved in the ubiquitin-dependent proteolysis of p21 are upregulated [271]. Some authors showed that the oncogenic potential of p21 is activated when it accumulates in the cytoplasm, while the tumor suppressive functions were associated with its nuclear localization [272]. Thus, p21 is phosphorylated by AKT in the nucleus and then translocates into the cytoplasm, where it accumulates and exerts anti-apoptotic effects [272]. In gastric cancer, nuclear p21 inhibits, while cytoplasmic p21 promotes cancer cell migration and invasion [270]. In BC patients, cytoplasmic overexpression of phospho-p21, HER2/*neu* receptor tyrosine kinase, and phospho-AKT have been associated with worse overall survival [273].

## 3. Discussion

Post-translational modifications (PTMs) of proteins assure a dynamic and adapting interface between oncogenic mutations and environmental stressors, on the one hand, and cancer cell structure, functioning, and behavior. Aberrant PTMs can be considered as a specific group of enabling characteristics of cancer as long as they orchestrate all malignant modifications and variability in the proteome of cancer cells, cancer-associated cells, and TME. In this review, we summarized and analyzed a wide spectrum of PTMs of proteins involved in all regulatory mechanisms that drive tumorigenesis, genetic instability, epigenetic reprogramming, all events of the metastatic cascade, angiogenesis, immune response, tumor-associated microbiome, and metabolism rewiring.

Sustaining proliferative signaling is a crucial process for carcinogenesis and tumor progression. We analyzed PTMs that occur in the most important biomarkers of cell proliferation, such as octamer-binding transcription factor 4 (OCT4), SRY-box transcription factor 2 (SOX2), and NANOG, cluster of differentiation CD24/44, cyclin-dependent kinases (CDKs), Ki-67 nuclear biomarker used by histopathologists to identify proliferating cells by immunohistochemical techniques, and specificity protein 1 (SP1), known to modulate cancer cell proliferation through its translocation during the cell cycle progression. The evading of anti-growth signaling is also an important hallmark of cancer cells, and PTMs of PTEN, RB1 protein, and YAP/TAZ are important in the modulation of growth signaling. Reversible epigenetics modifications sustain nonmutational epigenetic reprogramming, including DNA hypo- or/and hypermethylation, PTMs of proteins, including histone and non-histone proteins, and noncoding RNAs (ncRNAs) affect gene expression in cancer cells by alteration of chromatin and DNA accessibility without changes on DNA sequences. We analyzed the PTM of DNA methyltransferases aberrantly expressed in cancer, as well as aberrant histone PTMs. Avoiding immune destruction is required for cancer progression. PTMs of the PD-1/PD-L1 axis, including ubiquitination/deubiquitination, glycosylation, phosphorylation, palmitoylation, SUMOylation, NEDDylation, and acetylation, play a crucial role in avoiding immune destruction signaling pathway. Enabling replicative immortality is also a crucial hallmark of cancer cells, based on telomeres activity to maintain genomic integrity. Phosphorylation, ubiquitination, and SUMOylation have been identified as important PTMs that modulate the activity of human telomerase reverse transcriptase (hTERT) that maintain telomerase integrity and activity, as well as telomere elongation and tumorigenesis. Chronic inflammation promotes tumor progression and treatment resistance, while induction of acute inflammation leads to anti-tumor immune responses. Thus, PTMs of NF-kB and STAT family members induce their nuclear translocation and activation of transcription of target genes. Recently, dysbiotic microbiota was recognized as an important hallmark of cancer. Phosphorylation of c-Jun N-terminal kinase (JNK) and signal transducer and activator of transcription 3 (STAT3) induced by dysbiotic gut microbiome can accelerate tumor growth.

The development of the metastatic cascade is also a crucial hallmark of malignancy. PTMs of tight junction proteins, including epithelial cell adhesion molecule (EpCAM) and claudins, cytoskeletal proteins (vimentin, actins, and tubulins), HIF1α, EMT-TFs, and matrisome proteins (collagen, fibronectin, and metalloproteinases) modulate invasion and metastasis of cancer. PTMs of GLI1 protein regulate its intracellular trafficking and the transcriptional activity of the sonic hedgehog signaling pathway that promotes angiogenesis. Cellular senescence acts as a tumor-suppressor mechanism by preventing the growth of damaged cells, but it can also play, under certain conditions, a tumor-promoting role by building an inflammatory microenvironment. p21 and p16 PTMs are involved in the modulation of pathways related to cellular senescence. Genomic instability is also a hallmark of most cancer cells during cell division due to mutations in DNA damage repair genes or mitotic checkpoint genes, which predispose cells to malignant transformation. PTMs of tumor-suppressor p53, BRCA1, and GATA3 are involved in genome instability and mutation. Apoptosis is a major form of programmed cell death or cell suicide, and evasion of apoptosis or aberrant apoptosis is a hallmark of cancer that results in uncontrolled cell proliferation in tumors. PTMs of caspases, the oncogenic Bcl-2 family proteins, and pro-apoptotic BAX proteins are involved in resisting cell death. Cancer can be characterized as a metabolic disease and metabolic reprogramming is considered the most important emerging hallmark of tumor cells. We emphasized the role of several PTMs of hexokinases, pyruvate kinase M2, fatty acid synthase, nucleolin, and aldolase B involved in deregulating cellular metabolism. Finally, cell plasticity refers to the ability of cells to be reprogrammed, enabling them to acquire novel molecular, phenotypic, and functional traits that allow for tumor progression, metastasis, and therapy resistance. We emphasized that PTMs of proteins involved in activating invasion and metastasis also play a key role in unlocking phenotypic plasticity in cancer cells.

## 4. Conclusions

All hallmarks and enabling characteristics of cancer can develop due to aberrant PTMs of proteins, which modulate gene transcription, intracellular and extracellular signaling, protein size, activity, stability and localization, trafficking, secretion, intracellular protein degradation or half-life, and protein–protein interactions (PPIs). PTMs associated with cancer can be exploited to better understand the underlying molecular mechanisms of this heterogeneous and chameleonic disease, find new biomarkers of cancer progression and prognosis, personalize oncotherapies, and discover new targets for drug development. PTMs are involved in the dynamics of protein localization and can explain the false results that could be obtained by classic immunohistochemistry. Moreover, PTM quantification is also necessary to explain the dynamic and integrative nature of signaling pathways, as well as for understanding the functional role of these transient modifications of proteins in cancer cells.

Recent advances in mass spectrometry-based proteomics, especially based on mass spectrometry imaging (MSI), could be useful to deeply understand the role of PTMs of proteins in patient tumor tissue samples in order to translate the results obtained through cultured cell lines and animal models. In conclusion, aberrant PTMs of proteins can be considered as determinants or/and enabling characteristics of cancer. On the other hand, PTMs of proteins can enhance anticancer mechanisms in the tumoral ecosystem or sustain the beneficial effects of oncologic therapies by destruction or inactivation of carcinogenic proteins or activation of tumor-suppressor proteins.

## Figures and Tables

**Table 1:** Roles of the most common PTMs of proteins in cancer.

PTMs of Proteins	Definition	Roles in Cancer	References
phosphorylation	attachment of a phosphate group to specific amino acid residues of a protein	PPIs, cell growth, proliferation, division, differentiation, apoptosis, cell signaling, angiogenesis, metastasis	[31,32,33]
methylation	transfer of active methyl group to target histone or non-histone proteins	protein activity, localization, signaling, regulator of MAPK, WNT, JAK-STAT, Hippo, p53, NF-kB signaling	[34]
ubiquitination/deubiquitination	tagging of specifically targeted proteins with ubiquitin for degradation, translocation, or activation/reverse process of ubiquitination	proteostasis (protein stability/activity/degradation/turnover), DDR, immune response, cancer cell stemness, proliferation via cell cycle progression, all cancer hallmarks (evading growth suppressors, evasion of apoptosis, reprogramming energy metabolism, unlocking phenotypic plasticity, polymorphic microbiomes, immune evasion, resisting cell death, tumor-promoting inflammation, senescent cells)	[35,36,37,38]
SUMOylation	covalent attachment of a SUMO molecule to a substrate protein	chromatin organization, DDR, transcription, protein trafficking, signal transduction, TME formation and reprogramming, carcinogenesis, immune responses, cell cycle progression, apoptosis, metastasis	[39,40]
NEDDylation	attachment of NEDD8 to a lysine residue of a protein substrate (cullins and non-cullins proteins)	tumor progression (proliferation), increased malignancy, cancer cell behavior, regulation of protein degradation, angiogenesis, modulation of ECM, regulation of T-cell functionality	[28,41,42]
glycosylation	addition of individual carbohydrates or whole oligosaccharides (N-glycans, O-glycans, and proteoglycans) to corresponding protein or lipid	protein folding, secretion, cell adhesion, intra- and intercellular trafficking, cancer progression, cancer cell stemness, EMT, avoiding immune destruction	[11,43]
O-linked-β-N-Acetylglucosaminylation (O-GlcNAcylation)	addition of N-acetylglucosamine (GlcNAc) onto the hydroxyl groups of the Ser or Thr residues of proteins	development, maturation, and functions of immune cells, signal transduction, transcription, cell division, metabolism, and cytoskeletal regulation	[44]
acylation	transfer of acyl groups from acyl-CoA donors to sidechain of lysine (glycine, cysteine, serine, and others) residues of proteins	protein stability, subcellular localization, enzyme activity, transcriptional activity, PPIs, protein-DNA interactions	[19,45]
acetylation (Kac)	attachment of acetyl group from acetyl-coenzyme A to a specific site on a polypeptide chain	gene expression, cell cycle progress, DDR, PPIs, protein-DNA interactions, cell proliferation	[18]
propionylation (Kpr)	addition of propionyl groups to specific amino acid residues in a protein	modification of protein structure and functions, gene regulation, metabolic pathway, cellular signaling networks	[46]
succinylation (ksucc)	attachment of a succinyl group to a lysine residue	tumorigenesis, transcriptional regulation of genes, energy metabolism	[22]
crotonylation (Kcr)	transfers of crotonyl group onto lysine residues using crotonyl-CoA as substrate under the action of crotonyltransferase	carcinogenesis, tumor progression	[21,47]
glutarylation (Kglu)	addition a five-carbon glutaryl group to lysine residue non-enzymatically driven by glutaryl-CoA	regulation of mitochondrial proteins and metabolic enzymes, regulation of gene expression	[19]
β-hydroxybutyrylation (Kbhb)	addition of β-hydroxybutyrate to lysine residue	attenuates ALDOB activity in ketogenic diet, inhibits mTOR, glycolysis, and suppresses cancer cell proliferation	[23,24]
2-hydroxybutyrylation (Khib)	addition of butanoate to lysine residues	chromatin structure, gene transcription, protein subcellular localization, PPIs, signal transduction, glucose metabolism, amino acid synthesis, cell proliferation	[48,49,50]
malonylation (Kmal)	attachment of a malonyl group to a lysine residue	protein activity, localization, PPIs	[20]
lactylation (Kla)	covalent attachment of lactic acid moieties to protein lysine residues	gene transcription/expression regulation, cancer progression	[51,52]
benzonylation (Kbz)	introduction of benzoyl group by replacement of H- attached to O or N or to aromatic nucleus, stimulated by sodium benzoate through benzoyl-CoA generation	gene transcription regulation	[53]
S-palmitoylation/S-acylation	reversible attachment of fatty acids onto cysteine residue	protein localization, membrane affinity, stability, accumulation, secretion, and function, PPIs; cell proliferation	[10,54]
S-nitrosylation	addition of a nitrosyl group to the reactive thiol group of cysteine to form S-nitrosothiol	protein stability and turnover, steroid synthesis, transcription regulation, DDR, cellular growth, apoptosis, and redox regulation	[29]
citrullination/arginine deimination	converts arginine residues in proteins to citrulline; human citrullinome include VIM, ACTs, COL, FN, CKs, TUBs, and histones	protein folding, PPIs, regulation of apoptosis and differentiation, promotion of EMT, and metastasis	[8,55,56,57]

Abbreviations: ACTs—actins; ALDOB—aldolase B; CKs—cytokeratins; COL—collagen; ECM—extracellular matrix; EMT-epithelial-to-mesenchymal transition; DDR—DNA damage repair; FN—fibronectin; mTOR—mechanistic/mammalian target of rapamycin; NEDD8—neural precursor cell expressed developmentally downregulated protein 8; PPIs—protein–protein interactions; PTMs—post-translational modifications of proteins; SUMO—small ubiquitin-related modifier; TME—tumor microenvironment, TUBs—tubulins; VIM—vimentin.
